# Effects of Intensive Systolic Blood Pressure Control on Kidney Outcomes in Patients With and Without CKD: A Post Hoc Analysis of SPRINT and ACCORD‐BP Trials

**DOI:** 10.1111/1753-0407.70162

**Published:** 2025-10-21

**Authors:** Xiaoli Xu, Xuan Zhao, Yi Ding, Xianglin Wu, Qiuyu Cao, Kan Wang, Yu Xiang, Siyu Wang, Xiaoyun Zhang, Min Xu, Tiange Wang, Zhiyun Zhao, Yuhong Chen, Jieli Lu, Yufang Bi, Mian Li, Yu Xu

**Affiliations:** ^1^ Department of Endocrine and Metabolic Diseases, Shanghai Institute of Endocrine and Metabolic Diseases, Ruijin Hospital Shanghai Jiao Tong University School of Medicine Shanghai China; ^2^ National Clinical Research Center for Metabolic Diseases (Shanghai), Key Laboratory for Endocrine and Metabolic Diseases of the National Health Commission of the PR China, Shanghai Key Laboratory for Endocrine Tumor, State Key Laboratory of Medical Genomics, Ruijin Hospital Shanghai Jiao Tong University School of Medicine Shanghai China

**Keywords:** albuminuria, blood pressure control, chronic kidney disease, estimated glomerular filtration rate, renal failure

## Abstract

**Background:**

The effects of intensive blood pressure (BP) control on adverse kidney outcomes remain undetermined.

**Methods:**

This post hoc analysis included the Systolic Blood Pressure Intervention Trial (SPRINT) participants and the Action to Control Cardiovascular Risk in Diabetes Blood Pressure (ACCORD‐BP) participants receiving standard glucose‐lowering treatment and satisfying SPRINT eligibility criteria. The risks of kidney events between intensive (systolic BP < 120 mmHg) and standard (systolic BP < 140 mmHg) controls in participants with or without baseline chronic kidney disease (CKD) were compared using Cox proportional hazards models.

**Results:**

A total of 10,946 participants (2724 with CKD and 8222 without CKD) were included. During the intervention and post‐intervention periods, the risk of renal failure was either reduced by intensive BP control in participants with CKD (HR = 0.46; 95% CI = 0.22–0.97) or was not significantly different between intensive and standard BP control in participants without CKD (HR = 0.88, 95% CI = 0.52–1.49; *p*_interaction_ = 0.128). The risk of ≥ 30% reduction in estimated glomerular filtration rate (eGFR) was increased and the risk of albuminuria was decreased by intensive BP control in participants with or without CKD. Intensive BP control increased the risk of CKD progression to moderate‐ or high‐risk category, but not to very‐high risk category according to the Kidney Disease Improving Global Outcomes (KDIGO) risk categories.

**Conclusions:**

The intensive BP control might increase the risk of mild CKD progression but not of more advanced CKD progression or renal failure compared with the standard BP control.

## Graphical abstract

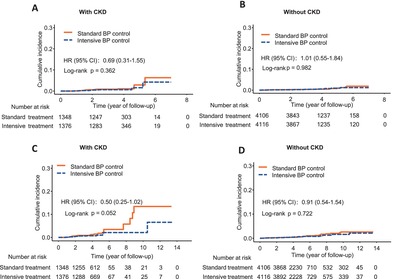

**Summary**

- The intensive BP control does not increase the risk of long‐term renal failure in participants with CKD or without CKD.
- The intensive BP control may increase the risk of a ≥ 30% reduction in eGFR and the risk of mild CKD progression, but it does not increase the risk of more advanced CKD progression, and it may decrease the risk of albuminuria.
- These findings need confirmation through continued follow‐up of trial participants or randomized trials with primary kidney outcomes.

## 1 Introduction

Hypertension leads to a progressive decline in kidney function, which in turn worsens blood pressure (BP) control [1]. Although hypertension is generally considered an important modifiable risk factor, the effect of intensive BP control on renal outcomes remains controversial [2]. The Systolic Blood Pressure Intervention Trial (SPRINT) reported that the intensive BP control induced a significant decline of estimated glomerular filtration rate (eGFR) in people without diabetes [3, 4, 5]. This was consistent with the findings from the Action to Control Cardiovascular Risk in Diabetes Blood Pressure Trial (ACCORD‐BP) in patients with type 2 diabetes [5, 6]. However, the risk of albuminuria was reduced by intensive BP control in these trials [4, 6]. The diverged effects on eGFR and albuminuria raise the question of whether intensive BP control was harmful or beneficial with regard to long‐term kidney outcomes.

Although the SPRINT study reported an increased risk of incident chronic kidney disease (CKD) defined by eGFR changes, it also revealed that the intensive BP control did not increase the risk of sustained large eGFR decline (≥ 50%) or long‐term dialysis in participants with CKD [4]. Recently, a pooled analysis of four randomized controlled trials (SPRINT, ACCORD‐BP, African American Study of Kidney Disease and Hypertension [AASK], and Modification of Diet in Renal Disease [MDRD] study) reported a lower risk of kidney replacement therapy in the intensive BP control group among CKD patients without acute eGFR decline during the early months of treatment [7]. However, the effect of intensive BP control in general on the risk of kidney replacement therapy remained unclear due to a limited number of events in previous trials.

Therefore, taking advantage of the increased statistical power by combining participants from SPRINT and ACCORD‐BP trials, as well as events that occurred during intervention and post‐intervention observational follow‐up periods, we performed a pooled analysis of participants from the two trials to determine the effects of intensive BP control on the risk of adverse kidney outcomes.

## 2 Materials and Methods

### 2.1 Data Acquisition

All data used for this study analysis has been publicly available at the National Heart, Lung, and Blood Institute's Biologic Specimen and Data Repository Information Coordinating Center (BioLINCC). We accessed SPRINT and ACCORD trial datasets as requested at https://biolincc.nhlbi.nih.gov.

### 2.2 Study Design and Study Participants

The individual data from the SPRINT and ACCORD‐BP trials were used for the current analysis. The trials are registered with ClinicalTrials.gov, numbers NCT01206062 (SPRINT) and NCT00000620 (ACCORD). Details of the study protocol and study procedures of the SPRINT and ACCORD‐BP trials have been published elsewhere [4, 6]. Briefly, the SPRINT was a multicenter randomized clinical trial in which 9361 eligible hypertensive patients with an increased risk for cardiovascular disease (CVD) but without type 2 diabetes or prior stroke were assigned to receive an intensive (target systolic BP [SBP] < 120 mmHg) or standard (target SBP < 140 mmHg) BP control treatment. The ACCORD‐BP used the same intensive or standard BP control treatment by randomization of 4733 eligible hypertensive patients, except that ACCORD‐BP participants had type 2 diabetes and were assigned to receive an intensive (glycated hemoglobin [HbA1c] goal < 6.0%) or standard (HbA1c goal 7.0%–7.9%) glucose‐lowering treatment at the same time (2 × 2 factorial design). To ensure comparability and consistency with the SPRINT trial, we applied the SPRINT inclusion criteria to the ACCORD‐BP trial participants (i.e., SPRINT‐eligible ACCORD‐BP participants). Specifically, we considered ACCORD‐BP participants to be SPRINT‐eligible if they were aged 50 years or older and met at least one of the following criteria: being 75 years or older, having a history of cardiovascular disease, presenting a 10‐year risk of atherosclerotic cardiovascular disease (Framingham risk score) of over 15%, or having a history of CKD. Additionally, we limited our analysis to participants in the standard glucose‐lowering treatment group, as intensive glucose control was not recommended by the American Diabetes Association (ADA) guideline [8] and to avoid potential interactive effects between blood pressure and glucose control interventions. This approach is consistent with previous studies [9, 10] that have applied similar criteria to the ACCORD‐BP population. We pooled the SPRINT and SPRINT‐eligible ACCORD‐BP datasets during the intervention and post‐intervention observational follow‐up periods for the current post hoc analysis (Figure S1).

### 2.3 Procedures and Outcomes

The intervention of the SPRINT trial was stopped early on August 20, 2015, after a median follow‐up of 3.3 years because of the significant benefit observed for major CVD events in the intensive BP control group. SPRINT participants were followed up after intervention until July 29, 2016, with a median intervention plus post‐intervention observational period of 3.9 years. Serum creatinine was measured at baseline, at 1, 3, 6, and every 6 months thereafter during intervention, and at the end of the post‐intervention observational period. Urinary albumin and urinary creatinine were measured at baseline, at 6, 12, 24, and 48 months during intervention, and at the end of the post‐intervention observational period.

The intervention of the ACCORD‐BP trial was completed in June 2009 after a mean follow‐up of 4.7 years. Among 2060 SPRINT‐eligible ACCORD‐BP participants, 1735 were followed up after intervention until April 30, 2015, with a median intervention plus post‐intervention observational period of 9.2 years. Serum creatinine was measured at baseline, at 4, 8, 12, and every 12 months thereafter, and the closeout visit during intervention. Urinary albumin and urinary creatinine were measured at baseline, at 24, 48 months, and the closeout visit during intervention. During the post‐intervention observational period, serum creatinine, urinary albumin, and urinary creatinine were also measured at the first and the last clinic visits.

For both trials, the four‐variable modification of diet in renal disease (MDRD) equation was used to calculate eGFR levels using serum creatinine concentration, to be consistent with previous publications of the SPRINT and ACCORD‐BP trials [11, 12]. Urinary albumin‐to‐creatinine (UACR) was calculated as urinary albumin concentration (mg/L) divided by urinary creatinine concentration (g/L). The adverse kidney outcomes in the current study included renal failure, eGFR decline, albuminuria, and CKD risk progression according to the Kidney Disease Improving Global Outcomes (KDIGO) risk categories [13] (Figure S2). Renal failure was defined as the onset of dialysis or transplantation in the SPRINT trial, and a composite occurrence of serum creatinine concentration > 3.3 mg/dL, initiation of dialysis or renal transplantation in the ACCORD‐BP trial. For the outcome of eGFR decline, the ≥ 50%, ≥ 40%, and ≥ 30% declines in eGFR from baseline were used. For the outcome of albuminuria, incident albuminuria was defined as a doubling of UACR from a value < 10 mg/g to a value of > 10 mg/g, microalbuminuria was defined by UACR > 30 mg/g, and macroalbuminuria was defined by UACR > 300 mg/g. All definitions using eGFR or UACR levels required a confirmatory value in the next available laboratory check.

### 2.4 Statistical Analysis

Participants with an eGFR level < 60 mL/min/1.73 m^2^ at baseline were defined as participants with CKD [4]. All analyses were conducted according to baseline CKD status. Baseline characteristics were summarized as numbers (percentages) for categorical variables, means ± standard deviations (SDs) or medians (interquartile ranges) for continuous variables. Student's *t*‐tests were used for comparisons of continuous variables, and chi‐square tests were used for comparisons of categorical variables.

Incidence rates of each outcome were calculated per 1000 person‐years of follow‐up. For the outcome of renal failure, follow‐up time was determined by the time from randomization to the onset of renal replacement therapy, a serum creatinine concentration > 3.3 mg/dL (ACCORD), death, or the participant's date of last follow‐up, whichever was the shortest. For the outcome of eGFR decline, follow‐up time was determined by the shortest time from randomization to the onset of eGFR decline, death, or the participant's date of last creatinine measurement. For the outcome of albuminuria, follow‐up time was determined by the shortest time from randomization to the onset of albuminuria, death, or the participant's date of last UACR measurement. For the outcome of CKD risk progression, follow‐up time was determined by the shortest time from randomization to the onset of CKD risk progression, death, or the participant's date of last creatinine and UACR measurements. For laboratory values with missing dates, follow‐up time was imputed with the median value of the corresponding visit. Kaplan–Meier curves were constructed to depict the cumulative incidence of renal failure and log‐rank tests were used to compare the cumulative incidence between the intensive and standard BP control groups. Hazard ratios (HRs) and 95% confidence intervals (95% CIs) for each outcome comparing intensive and standard BP control groups were calculated using Cox proportional hazards regression models adjusted for baseline age, sex, race, history of cardiovascular disease, tobacco use, body‐mass index, diabetes status, eGFR, UACR, SBP, and total cholesterol. We repeated the analysis in SPRINT participants and SPRINT‐eligible ACCORD‐BP participants, separately as the sensitivity analysis. All the analyses were conducted over the period of intervention and the period of intervention plus post‐intervention observational follow‐up, respectively, to explore the long‐term legacy effects of BP control on kidney outcomes.

The statistical analyses were performed with R version 4.2.3 and SAS version 9.4. A *p* value < 0.05 was considered significant.

## 3 Results

### 3.1 Participant Characteristics

There were 10,946 participants in the current study, including 2724 participants with CKD and 8222 participants without CKD. The mean age of the study participants was 67.1 years, and 36.9% were women. Patients with CKD were older, more likely to be women or white, and had lower eGFR and higher UACR levels compared with those without CKD (Table S1). Baseline characteristics were similar between intensive and standard BP control groups in participants with and without CKD (Table 1). The mean SBP achieved after randomization was significantly lower in the intensive BP control group compared with the standard BP control group during intervention. As expected, the mean SBP in both groups tended to converge during the post‐intervention observational period (Figure S3).

**TABLE 1:** Baseline characteristics of the study participants.

Characteristics	Participants with CKD at baseline		*p*	Participants without CKD at baseline		*p*
	Intensive	Standard		Intensive	Standard	
	(*n* = 1376)	(*n* = 1348)		(*n* = 4116)	(*n* = 4106)	
Age, years	71.5 (9.0)	71.6 (9.4)	0.782	65.6 (8.6)	65.6 (8.6)	0.803
Female, *n* (%)	567 (41.2)	542 (40.2)	0.623	1479 (35.9)	1451 (35.3)	0.589
Race or ethnic group, *n* (%)			0.180			0.571
Black	330 (24.0)	307 (22.8)		1239 (30.1)	1288 (31.4)	
Hispanic	100 (7.3)	99 (7.3)		448 (10.9)	431 (10.5)	
White	908 (66.0)	920 (68.2)		2270 (55.2)	2241 (54.6)	
Other	38 (2.8)	22 (1.6)		159 (3.9)	146 (3.6)	
History of CVD, *n* (%)	345 (25.1)	350 (26.0)	0.624	899 (21.8)	895 (21.8)	0.983
Framingham risk score, %	27.9 (14.8)	28.1 (14.7)	0.700	27.1 (14.2)	26.9 (13.8)	0.480
Current smoking, *n* (%)	108 (7.8)	117 (8.7)	0.473	626 (15.2)	563 (13.7)	0.058
BMI, kg/m^2^	29.7 (5.8)	29.6 (5.8)	0.393	30.6 (5.8)	30.5 (5.7)	0.583
SBP, mmHg	139.2 (16.0)	139.4 (16.1)	0.662	139.9 (15.8)	140.2 (15.3)	0.334
DBP, mmHg	75.0 (12.1)	74.6 (12.2)	0.390	78.7 (11.4)	78.8 (11.4)	0.756
Fasting plasma glucose, mg/dL	102.7 (27.7)	102.3 (27.1)	0.714	117.2 (44.4)	116.5 (44.0)	0.503
Total cholesterol, mg/dL	187.3 (41.2)	185.6 (41.0)	0.296	192.8 (42.8)	191.3 (41.0)	0.095
Triglycerides, mg/dL	112.0 (81.0, 156.0)	115.0 (83.0, 164.2)	0.039	113.0 (79.8, 165.0)	111.0 (80.0, 164.0)	0.739
LDL, mg/dL	109.1 (35.2)	106.3 (34.0)	0.030	113.9 (36.4)	112.7 (35.0)	0.121
HDL, mg/dL	52.2 (14.8)	51.8 (14.8)	0.490	51.3 (14.2)	51.3 (14.6)	0.777
eGFR, mL/min/1.73 m^2^	48.0 (9.4)	48.0 (9.4)	0.930	84.5 (22.1)	84.2 (18.3)	0.407
UACR, mg/g	13.3 (6.6, 43.9)	14.1 (6.2, 48.1)	0.688	9.7 (5.8, 21.0)	9.3 (5.7, 21.0)	0.456
UACR categories, *n* (%)			0.596			0.896
UACR < 30 mg/g	935 (68.0)	895 (66.4)		3331 (80.9)	3322 (80.9)	
UACR 30–300 mg/g	344 (25.0)	360 (26.7)		690 (16.8)	683 (16.6)	
UACR > 300 mg/g	97 (7.0)	93 (6.9)		95 (2.3)	101 (2.5)	
KDIGO risk categories, *n* (%)			0.706			0.896
Low	0 (0.0)	0 (0.0)		3331 (80.9)	3322 (80.9)	
Moderate	688 (50.0)	655 (48.6)		690 (16.8)	683 (16.6)	
High	431 (31.3)	427 (31.7)		95 (2.3)	101 (2.5)	
Very high	257 (18.7)	266 (19.7)		0 (0.0)	0 (0.0)	
Trial, *n* (%)			0.373			0.902
ACCORD‐BP	92 (6.7)	78 (5.8)		949 (23.1)	941 (22.9)	
SPRINT	1284 (93.3)	1270 (94.2)		3167 (76.9)	3165 (77.1)	

*Note:* Categorical variables are shown as numbers (%) and continuous variables are shown as means ± standard deviations (SDs) or medians (interquartile ranges). Student's *t*‐tests were used for comparisons of continuous variables and chi‐square tests were used for comparisons of categorical variables.

Abbreviations: BMI, body‐mass index; CKD, chronic kidney disease; CVD, cardiovascular disease; DBP, diastolic blood pressure; eGFR, estimated glomerular filtration rate; HDL, high density lipoprotein; LDL, low density lipoprotein; SBP, systolic blood pressure; UACR, urine albumin‐to‐creatinine ratio.

### 3.2 Intervention Period

**FIGURE 1:**
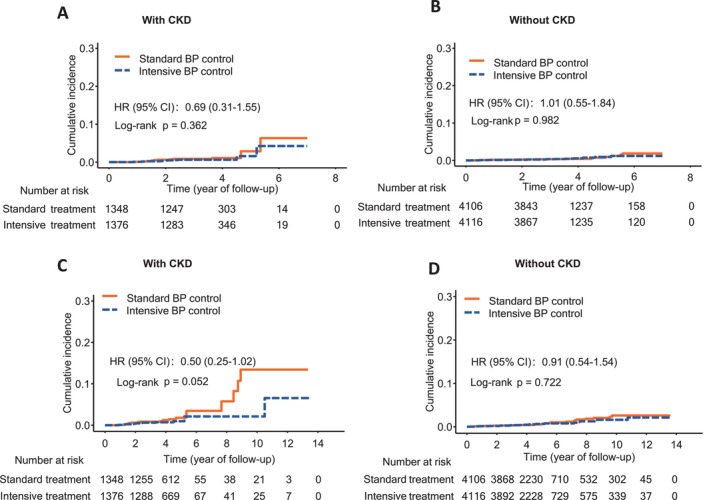
Kaplan–Meier curves of the cumulative incidence of renal failure by BP control groups in participants with or without CKD at baseline. (A) Cumulative incidence during the intervention period in participants with CKD at baseline. (B) Cumulative incidence during the intervention period in participants without CKD at baseline. (C) Cumulative incidence during the intervention and post‐intervention observational periods in participants with CKD at baseline. (D) Cumulative incidence during the intervention and post‐intervention observational periods in participants without CKD at baseline. Hazard ratios and 95% confidence intervals were not adjusted.

During the intervention period of a median 3.45 years, 24 participants with CKD developed renal failure (10 in the intensive BP control group and 14 in the standard BP control group) and 42 participants without CKD developed renal failure (21 in the intensive BP control group and 21 in the standard BP control group) (Table 2). Kaplan–Meier curves and log‐rank tests revealed no significant difference in cumulative incidence of renal failure between the two BP control groups (Figure 1A,B). No statistically significant difference was found for renal failure between intensive and standard BP controls in participants with CKD (HR 0.64 [95% CI 0.27–1.53]; *p* = 0.313) and without CKD (HR 0.99 [95% CI 0.54–1.82]; *p* = 0.986) (Table 2).

**TABLE 2:** Effects of intensive vs. standard BP control on kidney outcomes in participants with and without CKD during the intervention period.

	Participants with CKD at baseline		HR^a^ (95% CI)	*p*	Participants without CKD at baseline		HR^a^ (95% CI)	*p*	*p* for interaction
	Intensive	Standard			Intensive	Standard			
	Events (events/1000 person‐years)	Events (Events/1000 person‐years)			Events (events/1000 person‐years)	Events (events/1000 person‐years)			
Renal failure^b^	10 (2.17)	14 (3.14)	0.64 (0.27–1.53)	0.313	21 (1.43)	21 (1.44)	0.99 (0.54–1.82)	0.986	0.351
eGFR decline^c^									
≥ 30% reduction	107 (27.06)	58 (15.23)	1.79 (1.30–2.48)	< 0.001	451 (36.20)	186 (14.22)	2.59 (2.18–3.08)	< 0.001	0.057
≥ 40% reduction	33 (8.05)	21 (5.44)	1.55 (0.89–2.70)	0.124	159 (12.10)	68 (5.09)	2.37 (1.78–3.16)	< 0.001	0.159
≥ 50% reduction	14 (3.38)	14 (3.61)	0.91 (0.42–1.96)	0.813	50 (3.73)	27 (2.01)	1.77 (1.10–2.85)	0.019	0.149
Albuminuria^d^									
Incident albuminuria	52 (14.22)	61 (17.99)	0.78 (0.54–1.13)	0.194	144 (11.94)	168 (13.90)	0.86 (0.69–1.08)	0.200	0.756
Microalbuminuria	51 (13.95)	97 (29.01)	0.46 (0.33–0.65)	< 0.001	110 (9.06)	152 (12.56)	0.71 (0.55–0.91)	0.006	0.065
Macroalbuminuria	26 (7.01)	45 (13.09)	0.50 (0.31–0.82)	0.005	19 (1.54)	46 (3.74)	0.39 (0.23–0.67)	< 0.001	0.518
CKD risk progression according to KDIGO risk categories^e^									
Low‐risk to moderate‐risk	—	—	—	—	358 (30.79)	266 (22.41)	1.41 (1.21–1.66)	< 0.001	—
Low‐risk to high‐risk	—	—	—	—	42 (3.42)	18 (1.46)	2.34 (1.35–4.07)	0.003	—
Low‐risk to very‐high‐risk	—	—	—	—	3 (0.24)	5 (0.41)	0.57 (0.13–2.40)	0.440	—
Moderate‐risk to high‐risk	103 (29.03)	64 (18.87)	1.54 (1.13–2.11)	0.007	54 (4.41)	65 (5.31)	0.79 (0.55–1.14)	0.213	0.008
Moderate‐risk to very‐high‐risk	10 (2.68)	9 (2.57)	1.09 (0.44–2.69)	0.855	8 (0.65)	8 (0.65)	1.04 (0.38–2.80)	0.945	0.914
High‐risk to very‐high‐risk	43 (11.73)	28 (8.10)	1.42 (0.88–2.29)	0.156	16 (1.30)	9 (0.73)	1.75 (0.74–4.14)	0.199	0.660

Abbreviations: eGFR, estimated glomerular filtration rate; UACR, urine albumin‐to‐creatinine ratio.

^a^ Adjusted for baseline age, sex, race, history of cardiovascular disease, tobacco use, body‐mass index, diabetes status, eGFR, UACR, SBP, and total cholesterol.

^b^ Renal failure was defined by the need for dialysis or transplantation in the SPRINT trial, and a composite occurrence of serum creatinine concentration > 3.3 mg/dL, initiation of dialysis, or renal transplantation in the ACCORD‐BP trial.

^c^ Reductions in eGFR were defined as ≥ 50%, ≥ 40%, and ≥ 30% decline in eGFR levels from the baseline, and confirmed by a next available official laboratory test.

^d^ Incident albuminuria was defined by a doubling of UACR from a value < 10 mg/g to a value of > 10 mg/g; microalbuminuria was defined by UACR > 30 mg/g; macroalbuminuria was defined by UACR ratio > 300 mg/g. Albuminuria definitions were confirmed by the next available official laboratory test.

^e^ Progression risk of CKD was classified based on eGFR and UACR according to KDIGO risk categories at baseline and follow‐up periods, and confirmed by the next available official laboratory test.

Furthermore, as shown in Table 2, a significantly higher risk of ≥ 30% eGFR decline was observed in the intensive BP control group compared with the standard BP control group in participants with or without CKD. For outcomes with more pronounced eGFR reduction such as ≥ 40% and ≥ 50% eGFR decline, risks were significantly higher in the intensive vs. standard BP control group in participants without CKD but not in participants with CKD. Meanwhile, risks of microalbuminuria and macroalbuminuria were significantly reduced by intensive BP control in participants with or without CKD. The intensive BP control significantly enhanced the progression from KDIGO moderate‐risk to high‐risk category in participants with CKD, and from low‐risk to moderate‐risk category or from low‐risk to high‐risk category in participants without CKD. The intensive BP control did not statistically increase the risk of progression to very‐high‐risk category in participants with or without CKD.

### 3.3 Intervention Plus Post‐Intervention Observational Period

During the intervention plus post‐intervention observational period of a median 4.04 years, 34 participants with CKD developed renal failure (12 in the intensive BP control group and 22 in the standard BP control group) and 56 participants without CKD developed renal failure (27 in the intensive BP control group and 29 in the standard BP control group) (Table 3). Kaplan–Meier curves and log‐rank tests revealed a difference with borderline significance in cumulative incidence of renal failure between the two BP control groups in participants with CKD (Figure 1C). In addition, the Cox regression analysis demonstrated a significant 54% risk reduction for renal failure by intensive BP control in participants with CKD (HR 0.46 [95% CI 0.22–0.97]; *p* = 0.041) (Table 3). No significant difference was observed between BP control groups in participants without CKD (HR 0.88 [95% CI 0.52–1.49]; *p* = 0.648) (Figure 1D, Table 3). However, there is no evidence of interaction by CKD status (*p*_interaction_ = 0.128).

**TABLE 3:** Effects of intensive versus standard BP control on kidney outcomes in participants with and without CKD during the intervention and post‐intervention observational periods.

	Participants with CKD at baseline		HR^a^ (95% CI)	*p*	Participants without CKD at baseline		HR^a^ (95% CI)	*p*	*p* for interaction
	Intensive	Standard			Intensive	Standard			
	Events (events/1000 person‐years)	Events (events/1000 person‐years)			Events (events/1000 person‐years)	Events (events/1000 person‐years)			
Renal failure^b^	12 (2.16)	22 (4.12)	0.46 (0.22–0.97)	0.041	27 (1.37)	29 (1.49)	0.88 (0.52–1.49)	0.648	0.128
eGFR decline^c^									
≥ 30% reduction	140 (28.98)	72 (15.38)	1.92 (1.44–2.55)	< 0.001	534 (33.79)	253 (15.11)	2.29 (1.97–2.66)	< 0.001	0.314
≥ 40% reduction	45 (8.92)	34 (7.13)	1.24 (0.79–1.95)	0.352	201 (11.82)	101 (5.85)	2.04 (1.60–2.59)	< 0.001	0.070
≥ 50% reduction	21 (4.12)	20 (4.16)	0.92 (0.49–1.73)	0.800	62 (3.55)	42 (2.40)	1.41 (0.94–2.09)	0.094	0.289
Albuminuria^d^									
Incident albuminuria	75 (15.53)	108 (24.17)	0.62 (0.46–0.83)	0.002	224 (13.46)	267 (16.17)	0.83 (0.69–0.99)	0.036	0.124
Microalbuminuria	88 (18.26)	139 (31.51)	0.55 (0.42–0.73)	< 0.001	183 (10.92)	235 (14.17)	0.76 (0.62–0.92)	0.004	0.079
Macroalbuminuria	44 (8.95)	65 (14.25)	0.60 (0.41–0.88)	0.008	43 (2.51)	60 (3.52)	0.68 (0.46–1.01)	0.054	0.605
CKD risk progression according to KDIGO risk categories^e^									
Low‐risk to moderate‐risk	—	—	—	—	505 (31.93)	375 (23.23)	1.42 (1.25–1.63)	< 0.001	—
Low‐risk to high‐risk	—	—	—	—	72 (4.23)	35 (2.05)	2.03 (1.35–3.05)	< 0.001	—
Low‐risk to very‐high‐risk	—	—	—	—	9 (0.52)	11 (0.64)	0.85 (0.35–2.08)	0.720	—
Moderate‐risk to high‐risk	138 (29.61)	85 (18.90)	1.55 (1.18–2.04)	0.002	76 (4.48)	82 (4.85)	0.88 (0.65–1.21)	0.439	0.010
Moderate‐risk to very‐high‐risk	21 (4.24)	13 (2.78)	1.47 (0.73–2.95)	0.282	18 (1.05)	17 (0.99)	1.00 (0.51–1.97)	0.987	0.399
High‐risk to very‐high‐risk	59 (12.16)	42 (9.13)	1.31 (0.88–1.96)	0.180	20 (1.17)	21 (1.23)	0.87 (0.46–1.67)	0.686	0.363

Abbreviations: eGFR, estimated glomerular filtration rate; UACR, urine albumin‐to‐creatinine ratio.

^a^ Adjusted for baseline age, sex, race, history of cardiovascular disease, tobacco use, body‐mass index, diabetes status, eGFR, UACR, SBP, and total cholesterol.

^b^ Renal failure was defined by the need for dialysis or transplantation in the SPRINT trial, and a composite occurrence of serum creatinine concentration > 3.3 mg/dL, initiation of dialysis, or renal transplantation in the ACCORD‐BP trial.

^c^ Reductions in eGFR were defined as ≥ 50%, ≥ 40%, and ≥ 30% decline in eGFR levels from the baseline, and confirmed by the next available official laboratory test.

^d^ Incident albuminuria was defined by a doubling of UACR from a value < 10 mg/g to a value of > 10 mg/g; microalbuminuria was defined by UACR > 30 mg/g; macroalbuminuria was defined by UACR ratio > 300 mg/g. Albuminuria definitions were confirmed by the next available official laboratory test.

^e^ Progression risk of CKD was classified based on eGFR and UACR according to KDIGO risk categories at baseline and follow‐up periods, and confirmed by the next available official laboratory test.

Table 3 also showed the outcomes of eGFR decline, albuminuria, and CKD risk progression defined by KDIGO risk categories; results were generally unchanged after the extension of the follow‐up period, except for incident albuminuria, which showed significant risk reduction by intensive BP control.

### 3.4 Sensitivity Analysis

We performed the sensitivity analysis among the SPRINT and SPRINT‐eligible ACCORD‐BP participants, separately. Generally, as shown in Tables S1–S4, results were similar to the main analyses. There was a risk reduction tendency for renal failure by intensive BP control during the intervention period for participants with CKD in both trials, and the risk reduction became statistically significant in the ACCORD participants after the extension of the follow‐up period.

## 4 Discussion

In this post hoc analysis, we combined the SPRINT and SPRINT‐eligible ACCORD‐BP participants to explore the association between intensive BP control and different types of adverse kidney outcomes during the intervention and post‐intervention observational periods among participants with and without CKD at baseline. We found that intensive BP control tended to reduce the risk of renal failure among participants with CKD, but the conclusion remains uncertain due to the limited number of events and the short duration of follow‐up. Intensive BP control increased the risk of a 30% reduction in eGFR, but not a 40% or 50% reduction in eGFR among participants with CKD, while it was associated with a significantly lower risk of albuminuria in participants with or without CKD. In addition, intensive BP control led to a mild progression of CKD risk, but not the progression to a very‐high CKD risk according to the KDIGO risk categories. Therefore, our findings indicated that intensive BP control might increase the risk of mild CKD progression but not the risk of more advanced CKD progression or renal failure compared with standard BP control.

Renal failure, which requires the use of replacement therapy (dialysis or kidney transplantation), represents the irreversible progression to renal parenchymal damage and the final stage of kidney disease [14]. Both SPRINT and ACCORD‐BP trials defined renal failure as an important kidney outcome [4, 11]. The SPRINT trial defined the development of renal failure as requiring chronic dialysis or kidney transplantation. The ACCORD‐BP trial defined the development of renal failure as a composite of serum creatinine concentration > 3.3 mg/dL, initiation of dialysis, or kidney transplantation. However, neither study reached a definitive conclusion on this outcome partly due to limited cases of events. In the current study, we defined renal failure according to the prespecified definitions in the trials. By combining data from both trials and extending the follow‐up to the post‐intervention period, we were able to examine a relatively long‐term effect of intensive vs. standard BP control on renal failure. In addition, because patients with and without CKD may require different BP control targets, we stratified the study participants according to their CKD status at baseline.

Recommendations by clinical guidelines of BP management in CKD patients have changed over the past decades. The Seventh Report of the Joint National Committee on Prevention, Detection, Evaluation, and Treatment of High Blood Pressure (JNC‐7) in 2003 recommended a treatment target of < 130/80 mmHg in patients with CKD [15] while the updated JNC‐8 in 2013 recommended a treatment target of < 140/90 mmHg [16]. The UK National Institute for Health and Care Excellence (NICE) recommended a BP target of < 140/90 mmHg in patients with a UACR < 70 mg/mol, and < 130/80 mmHg in those with a UACR > 70 mg/mol [17]. The updated KDIGO guideline in 2021 recommended that patients with CKD (not receiving dialysis) and hypertension should be treated to reach an SBP target of < 120 mmHg if tolerated, regardless of proteinuria status [18]. Consistent with the KDIGO guideline, Hypertension Canada also recommended an SBP target of < 120 mmHg [19]. Guidelines that tend to recommend a strict BP lowering target are because the SPRINT trial showed a significant reduction in major CVD events by intensive BP control and there was no difference among participants with and without CKD [12]. This recommendation is also based on the consideration that the risk of dying from CVD widely exceeds the risk of renal failure in CKD patients. Findings from our study provide further support for the KDIGO recommendation with regard to the end‐stage kidney outcome.

The BP treatment target in patients with CKD and other specific conditions also deserves attention. For patients after kidney transplantation, both KDIGO and the American College of Cardiology/American Heart Association (ACC/AHA) guidelines currently recommend a BP target of < 130/80 mmHg [18, 20], although whether lower BP targets would demonstrate benefits for the kidney and cardiovascular system remains unclear. For patients with CKD and type 2 diabetes, the latest findings from the Blood Pressure Control Target in Diabetes (BPROAD) trial showed that intensive BP control provided benefits with regard to the CVD outcome, but the effect on CKD progression was neutral compared with the standard BP control [21]. Previous studies revealed that diabetes aggravated kidney disease progression in hypertensive patients [22, 23]. The current study included the SPRINT‐eligible ACCORD‐BP participants who had type 2 diabetes, and it is noteworthy that the long‐term benefit of intensive BP control on renal failure was mainly addressed in those participants. Although the development of renal failure is rare, the significantly lower risk of renal failure in CKD participants given intensive BP control suggested that people with type 2 diabetes may be better suited for intensive BP control.

In addition to renal failure, we also included other adverse kidney outcomes reported in previous literature based on eGFR and UACR levels. These measurements have important implications for kidney and cardiovascular disease prediction [13, 24]. Previous clinical trials in non‐diabetic patients with CKD, such as the MDRD study [25] and the AASK study [11, 26], indicated that BP lowering led to a decrease in eGFR. Findings of the ACCORD‐BP and SPRINT trials also revealed that intensive BP control increased the risk of eGFR decline but reduced the risk of albuminuria [4, 5, 6]. In addition, a post hoc analysis of SPRINT revealed that the effects of intensive BP control on the increased risk of ≥ 40% eGFR decline were not modified by baseline albuminuria [27]. A meta‐analysis of 19 trials of more intensive vs. less intensive BP‐lowering treatment showed that more intensive treatment (mean BP levels of 133/76 mmHg) led to a significant risk reduction in albuminuria and did not increase the risk of ESKD compared with the less intensive treatment (mean BP levels of 140/81 mmHg) [3]. In the current study, the risk of a moderate (≥ 30%) reduction in eGFR was increased by intensive BP control in participants with and without CKD, while the risk of a severe (≥ 40% or ≥ 50%) reduction in eGFR by intensive BP control tended to be neutral compared with standard BP control, especially in participants with CKD. This was also demonstrated by the results of CKD risk progression according to KDIGO risk categories. The KDIGO risk classification utilizes both eGFR and UACR levels to define kidney risk categories. We found that intensive BP control may increase CKD progression to moderate or high KDIGO risk category but did not increase CKD progression to very‐high KDIGO risk category.

Studies have examined the interrelation of a reduced eGFR level and adverse clinical outcomes to elucidate whether there are true harms posed by the eGFR reduction during intensive BP control. Analyses of the SPRINT and the ACCORD‐BP trials have shown that intensive BP control does not increase the levels of kidney damage biomarkers despite lowering eGFR [28, 29]. Furthermore, a post causal mediation analysis of SPRINT indicated that there was no evidence that the reduction in eGFR owing to intensive BP control attenuated the cardiovascular or mortality benefits [30]. The pooled analysis of SPRINT, ACCORD‐BP, AASK, and MDRD study demonstrated that it was the acute decrease in eGFR during intensive or standard BP control, rather than the intensive BP control per se, that was associated with the increased risk of kidney replacement therapy [7]. Taken together, eGFR decline due to intensive BP control may predominantly reflect hemodynamic alterations in renal blood flow rather than intrinsic injury.

Our study has several strengths compared with previous studies. First, we added renal failure events during the post‐intervention observational period to those during the trial intervention period to increase the total number of renal failure events and statistical power. This is important because the effects of intensive BP control on hard renal outcomes have been uncertain due to the limited number of outcomes during the intervention. Previous studies have suggested that post‐trial follow‐up studies may offer valuable insights by identifying legacy effects [31, 32]. The current results indicated that intensive BP control might confer long‐term renal benefits, potentially through mechanisms such as reducing glomerular hyperfiltration and improving renal hemodynamics. Moreover, the cardiovascular legacy effects of intensive BP control, including improvements in vascular function and reductions in atherosclerotic burden, may also help improve renal outcomes. Second, we included all relevant CKD outcomes defined by different eGFR reduction levels, UACR levels, as well as risk progression according to the KDIGO risk categories using both UACR and eGFR levels, providing a comprehensive understanding of intensive BP control on kidney outcomes. Specifically, the inclusion of albuminuria in the analysis added to the previous literature. Although eGFR is widely recognized as a key indicator of kidney function, albuminuria has gained increasing attention in recent years because it is closely associated with the progression of kidney disease, particularly its early predictive role in kidney damage [18, 33].

There are also several limitations in the current study. First, it is post hoc and observational, and does not preserve the original randomization schema of the parent trials. Therefore, it suffers from inherent biases of an observational study such as confounding bias. Although a number of confounding factors were adjusted in the Cox models, there were still unmeasured and unknown confounding. In addition, we excluded ACCORD‐BP participants receiving intensive glucose‐lowering therapy because there might be interactions between glucose and BP interventions and the intensive target of a hemoglobin A1c < 6.0% is not recommended by current guideline [8]. However, this exclusion may lead to selection bias. Future studies should aim to account for these biases to elucidate the associations. Second, because renal failure is a rare clinical event, the accumulation of renal failure events is still limited even though data were pooled from 2 separate trials and the post‐intervention period was included for analysis. In addition, the follow‐up duration is short for the development of renal failure. Third, the number of participants is also limited, especially for participants with CKD and participants with type 2 diabetes. Larger, well‐powered studies with longer follow‐up durations, as well as collaborative efforts to use real‐world data, are needed to accumulate a sufficient number of renal failure events and fully elucidate the effects of intensive systolic BP control on hard renal outcomes. Finally, the current findings may not be applicable to other populations, such as individuals with low CVD risk.

In conclusion, the current study only found a potential increase in the risk of mild CKD progression, which did not translate into an increased risk of ultimate renal failure by intensive vs. standard BP control. In fact, the risk of renal failure might be reduced by intensive BP control in patients with CKD at baseline. Findings from the current study suggest that intensive BP control in CKD patients is safe and may potentially benefit long‐term kidney outcomes. However, because statistical testing results such as the *p* value for interaction of renal failure suggested limited statistical evidence of differential effects by CKD status, these findings should be interpreted with serious caution. Future confirmation through continued follow‐up of SRPINT and ACCORD participants and ideally by randomized trials of CKD patients receiving intensive vs. standard BP control for the prevention of adverse renal outcomes is warranted.

## Author Contributions

Conceptualization, formal analysis, methodology, and writing – original draft: Xiaoli Xu, Yufang Bi, Mian Li, and Yu Xu. Data analysis and interpretation: Xiaoli Xu, Xuan Zhao, Yi Ding, Xianglin Wu, Qiuyu Cao, Kan Wang, Yu Xiang, Siyu Wang, and Xiaoyun Zhang. Writing and editing: Xiaoli Xu, Xuan Zhao, Yi Ding, Xianglin Wu, Qiuyu Cao, Kan Wang, Yu Xiang, Siyu Wang, Xiaoyun Zhang, Min Xu, Tiange Wang, Zhiyun Zhao, Yuhong Chen, and Jieli Lu. Supervision or mentorship: Yufang Bi, Mian Li, and Yu Xu. All authors participated in the data analysis and preparation of the manuscript and approved the final manuscript for publication.

## Disclosure

Yufang Bi is the Editorial Board member of the Journal of Diabetes and the co‐authors of this article. To minimize bias, they were excluded from all editorial decision‐making related to the acceptance of this article for publication.

## Ethics Statement

SPRINT and ACCORD were both conducted in accordance with the principles of the Declaration of Helsinki, the International Conference on Harmonization Guidelines for Good Clinical Practice, and applicable regulations of all countries with participating study sites. Study protocols were reviewed and approved by Institutional Review Boards before the studies commenced. All patients provided written informed consent prior to participating in the study.

## Conflicts of Interest

The authors declare no conflicts of interest.

## Supporting information


**Data S1:** Supporting Information.

## Data Availability

The SPRINT and ACCORD data set can be applied from the National Heart, Lung and Blood Institute BioLlNCC data repository by visiting https://biolincc.nhlbi.nih.gov/home/.
