# Supplementary material for: Effects of Intensive Systolic Blood Pressure Control on Kidney Outcomes in Patients With and Without CKD: A Post Hoc Analysis of SPRINT and ACCORD‐BP Trials
Source: J Diabetes. 2025 Oct 21;17(10):e70162. doi: 10.1111/1753-0407.70162 (PMC12538231; doi:10.1111/1753-0407.70162)
Supplement: Supplementary file 1 — Data S1: Supporting Information. [file JDB-17-e70162-s001.docx]

**Supplementary Figures and Tables**

[Figure S1 Flowchart of study participants](#_Toc164683537)

Figure S2 KDIGO risk categories according to eGFR and UACR

[Figure S3 Systolic blood pressure levels among the study participants during intervention period and post-intervention observational period](#_Toc164683539)

[Table S1 Baseline characteristics of the study participants according to baseline CKD status](#_Toc164683540)

[Table S2 Effects of intensive vs. standard BP control on kidney outcomes in SPRINT participants with and without CKD during the intervention period](#_Toc164683541)

[Table S3 Effects of intensive vs. standard BP control on kidney outcomes in SPRINT participants with and without CKD during the intervention and post-intervention observational period](#_Toc164683542)

[Table S4 Effects of intensive vs. standard BP control on kidney outcomes in SPRINT eligible ACCORD-BP participants with and without CKD during the intervention period](#_Toc164683543)

[Table S5 Effects of intensive vs. standard BP control on kidney outcomes in SPRINT eligible ACCORD-BP participants with and without CKD during the intervention and post-intervention observational period](#_Toc164683544)

**Figure S1 Flowchart of study participants**


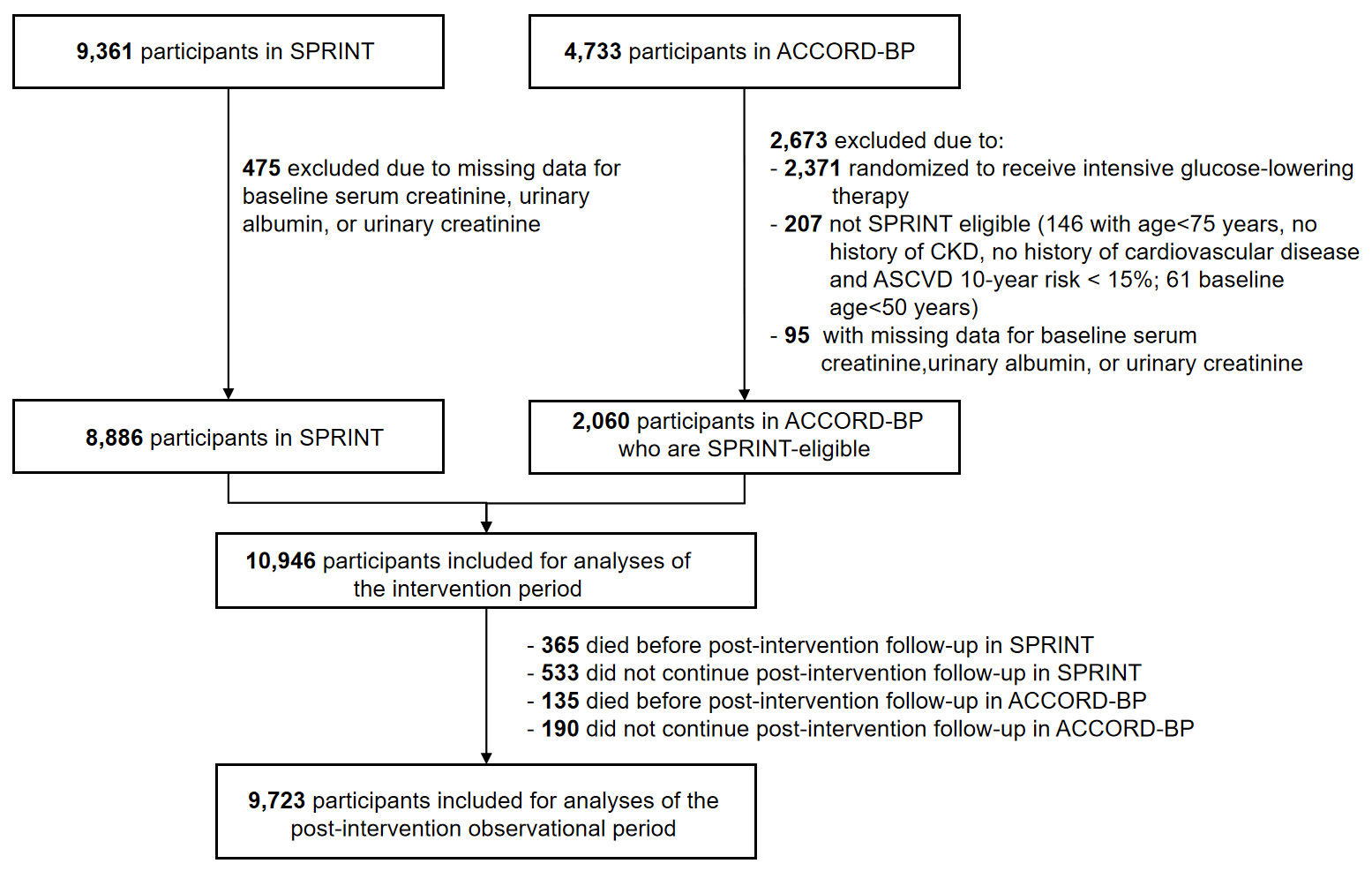


**Figure S2 KDIGO risk categories according to eGFR and UACR^1^**


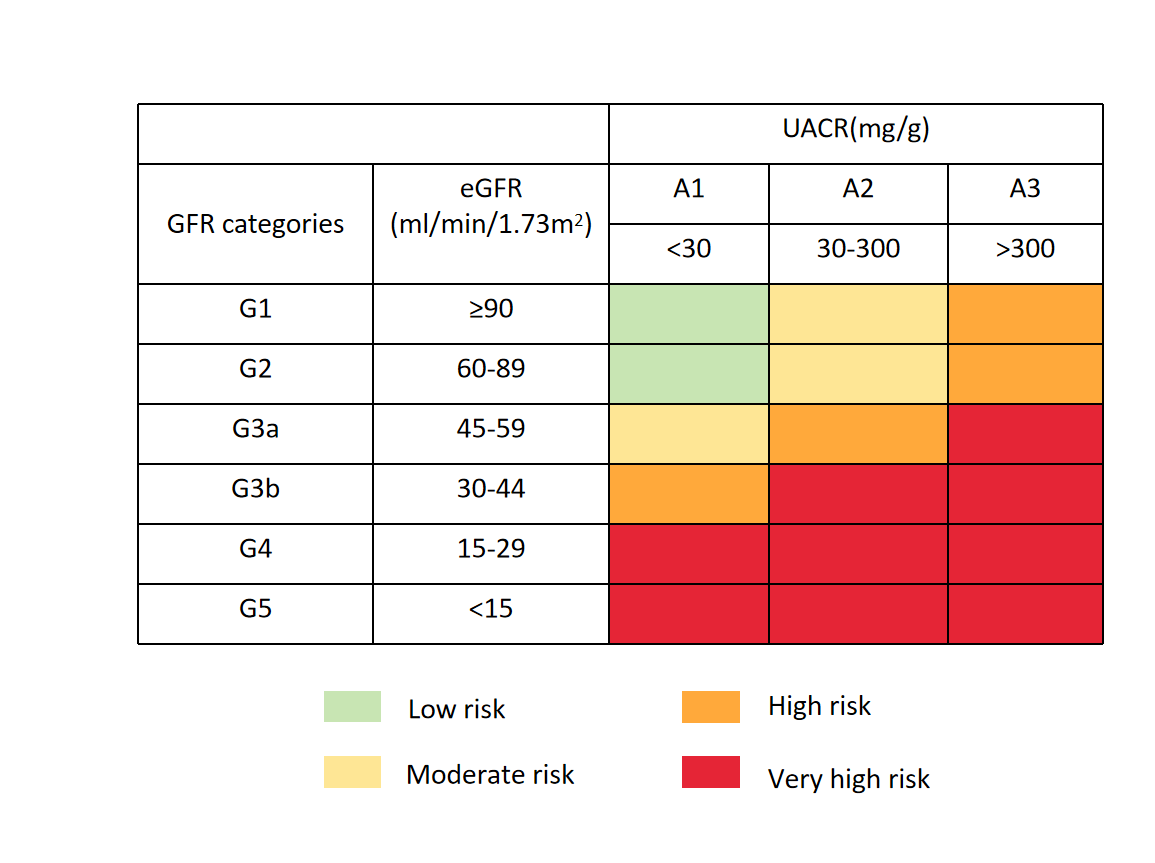


Abbreviations: eGFR, estimated glomerular filtration rate; UACR, urine albumin-to-creatinine ratio.

**References**

1.Stevens PE, Levin A. Evaluation and management of chronic kidney disease: synopsis of the kidney disease: improving global outcomes 2012 clinical practice guideline. *Ann Intern Med*. 2013;158(11):825-830. doi:10.7326/0003-4819-158-11-201306040-00007

**Figure S3 Systolic blood pressure levels among the study participants during intervention period and post-intervention observational period**


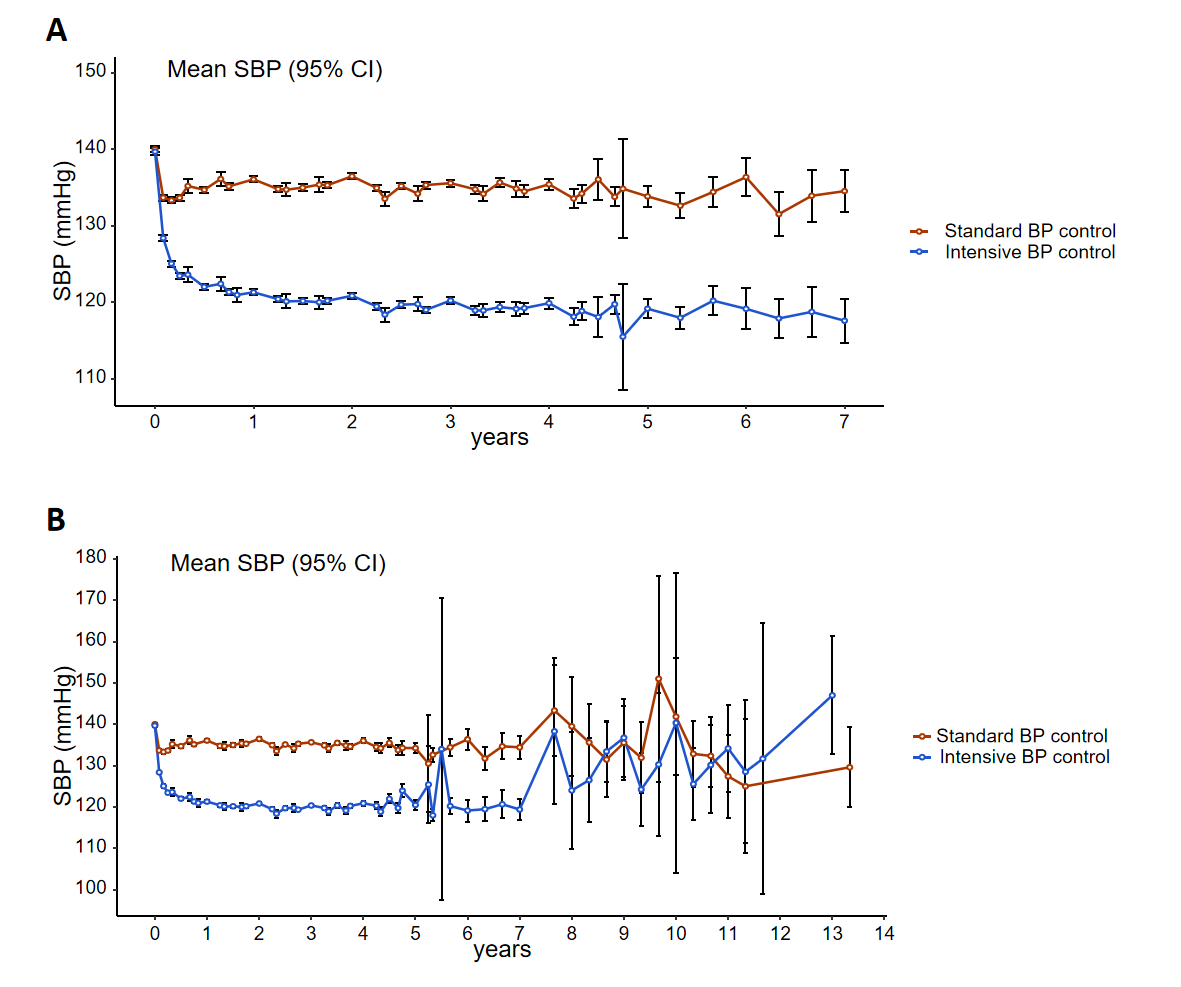


(A) The intervention period. (B) The intervention plus post-intervention observational period.

Abbreviation: SBP, systolic blood pressure; CI, confidence interval.

**Table S1 Baseline characteristics of the study participants according to baseline CKD status**

| **Characteristics** | **Overall**  (n=10,946) | **With CKD**  (n=2,724) | **Without CKD**  (n=8,222) | ***P* Value** |
| --- | --- | --- | --- | --- |
| Age, years | 67.1 (9.1) | 71.6 (9.2) | 65.6 (8.6) | <0.001 |
| Female, n (%) | 4039 (36.9) | 1109 (40.7) | 2930 (35.6) | <0.001 |
| Race or ethnic group, n (%) |  |  |  | <0.001 |
| Black | 3164 (28.9) | 637 (23.4) | 2527 (30.7) |  |
| Hispanic | 1078 (9.8) | 199 (7.3) | 879 (10.7) |  |
| White | 6339 (57.9) | 1828 (67.1) | 4511 (54.9) |  |
| Other | 365 (3.3) | 60 (2.2) | 305 (3.7) |  |
| History of CVD, n (%) | 2489 (22.7) | 695 (25.5) | 1794 (21.8) | <0.001 |
| Framingham risk score, % | 27.2 (14.2) | 28.0 (14.7) | 27.0 (14.0) | 0.001 |
| Current smoking, n (%) | 1414 (12.9) | 225 (8.3) | 1189 (14.5) | <0.001 |
| BMI, kg/m^2^ | 30.3 (5.8) | 29.6 (5.8) | 30.5 (5.7) | <0.001 |
| SBP, mmHg | 139.8 (15.7) | 139.3 (16.0) | 140.0 (15.5) | 0.032 |
| DBP, mmHg | 77.8 (11.7) | 74.8 (12.1) | 78.7 (11.4) | <0.001 |
| Fasting plasma glucose, mg/dL | 113.3 (41.2) | 102.5 (27.4) | 116.9 (44.2) | <0.001 |
| Total cholesterol, mg/dL | 190.7 (41.8) | 186.5 (41.1) | 192.1 (41.9) | <0.001 |
| Triglycerides, mg/dL | 112.0 (80.0, 164.0) | 114.0 (82.0, 160.0) | 112.0 (80.0, 164.0) | 0.618 |
| LDL, mg/dL | 111.9 (35.5) | 107.7 (34.6) | 113.3 (35.7) | <0.001 |
| HDL, mg/dL | 51.5 (14.5) | 52.0 (14.8) | 51.3 (14.4) | 0.029 |
| eGFR, mL/min/1.73 m^2^ | 75.3 (24.0) | 48.0 (9.4) | 84.3 (20.3) | <0.001 |
| UACR, mg/g | 10.2 (5.9, 25.8) | 13.8 (6.5, 45.0) | 9.5 (5.8, 21.0) | <0.001 |
| UACR categories, n (%) |  |  |  | <0.001 |
| UACR <30 mg/g | 8483 (77.5) | 1830 (67.2) | 6653 (80.9) |  |
| UACR 30-300 mg/g | 2077 (19.0) | 704 (25.8) | 1373 (16.7) |  |
| UACR >300 mg/g | 386 (3.5) | 190 (7.0) | 196 (2.4) |  |
| KDIGO risk categories, n (%) |  |  |  | <0.001 |
| Low | 6653 (60.8) | 0 (0.0) | 6653 (80.9) |  |
| Moderate | 2716 (24.8) | 1343 (49.3) | 1373 (16.7) |  |
| High | 1054 (9.6) | 858 (31.5) | 196 (2.4) |  |
| Very high | 523 (4.8) | 523 (19.2) | 0 (0.0) |  |
| Trial, n (%) |  |  |  | <0.001 |
| ACCORD-BP | 2060 (18.8) | 170 (6.2) | 1890 (23.0) |  |
| SPRINT | 8886 (81.2) | 2554 (93.8) | 6332 (77.0) |  |

Categorical variables are shown as numbers (%) and continuous variables are shown as means ± standard deviations (SDs) or medians (interquartile ranges). Student’s t-tests were used for comparisons of continuous variables and chi-square tests were used for comparisons of categorical variables.

Abbreviations: BMI, body mass index; CKD, chronic kidney disease; CVD, cardiovascular disease; DBP, diastolic blood pressure; eGFR, estimated glomerular filtration rate; HDL, high density lipoprotein; LDL, low density lipoprotein; SBP, systolic blood pressure; UACR, urine albumin-to-creatinine ratio.

**Table S2 Effects of intensive vs. standard BP control on kidney outcomes in SPRINT participants with and without CKD during the intervention period**

|  | **Participants with CKD at baseline** | |  |  | **Participants without CKD at baseline** | |  |  |  |
| --- | --- | --- | --- | --- | --- | --- | --- | --- | --- |
|  | **Intensive** | **Standard** | **HR^#^**  **（95% CI）** | ***P* Value** | **Intensive** | **Standard** | **HR^#^**  **（95% CI）** | ***P* Value** | ***P* for interaction** |
|  | Events  (Events/1,000  person-years) | Events  (Events/1,000  person-years) |  |  | Events  (Events/1,000  person-years) | Events  (Events/1,000  person-years) |  |  |  |
| ***Renal failure** | 7 (1.68) | 10 (2.44) | 0.51 (0.17-1.52) | 0.228 | 0 | 0 | - | - | - |
| **†eGFR decline** |  |  |  |  |  |  |  |  |  |
| ≥30% Reduction | 92 (25.78) | 48 (13.83) | 1.86 (1.31-2.64) | <0.001 | 196 (22.28) | 56 (6.20) | 3.63 (2.70-4.89) | <0.001 | 0.008 |
| ≥40% Reduction | 28 (7.60) | 17 (4.83) | 1.54 (0.84-2.83) | 0.165 | 54 (5.97) | 12 (1.32) | 4.61 (2.46-8.62) | <0.001 | 0.026 |
| ≥50% Reduction | 11 (2.96) | 11 (3.11) | 0.94 (0.40-2.21) | 0.885 | 12 (1.32) | 6 (0.66) | 2.04 (0.76-5.47) | 0.157 | 0.324 |
| **‡Albuminuria** |  |  |  |  |  |  |  |  |  |
| Incident albuminuria | 49 (15.07) | 53 (17.30) | 0.88(0.59-1.30) | 0.509 | 107 (13.56) | 123 (15.59) | 0.88 (0.68-1.14) | 0.337 | 0.954 |
| Microalbuminuria | 48 (14.77) | 88 (29.19) | 0.49 (0.35-0.70) | <0.001 | 62 (7.79) | 104 (13.16) | 0.59 (0.43-0.80) | <0.001 | 0.520 |
| Macroalbuminuria | 24 (7.28) | 37 (11.90) | 0.57 (0.34-0.96) | 0.033 | 4 (0.50) | 18 (2.24) | 0.20 (0.07-0.61) | 0.005 | 0.127 |
| **§CKD risk progression according to KDIGO risk categories** | | |  |  |  |  |  |  |  |
| Low-risk to moderate-risk | - | - | - | - | 267 (35.32) | 202 (26.18) | 1.43 (1.19-1.71) | <0.001 | - |
| Low-risk to high-risk | - | - | - | - | 22 (2.74) | 10 (1.24) | 2.32 (1.09-4.90) | 0.028 | - |
| Low-risk to very-high-risk | - | - | - | - | 0 | 2 (0.25) | - | - | - |
| Moderate-risk to high-risk | 91 (28.76) | 61 (20.01) | 1.45 (1.05-2.01) | 0.024 | 26 (3.24) | 31 (3.86) | 0.83 (0.49-1.41) | 0.494 | 0.073 |
| Moderate-risk to very-high-risk | 6 (1.81) | 8 (2.53) | 0.73 (0.25-2.11) | 0.557 | 2 (0.25) | 2 (0.25) | 0.98 (0.14-6.97) | 0.984 | 0.726 |
| High-risk to very-high-risk | 40 (12.26) | 22 (7.03) | 1.75 (1.04-2.95) | 0.036 | 5 (0.62) | 3 (0.37) | 0.55 (0.12-2.43) | 0.430 | 0.998 |

***** Renal failure was defined by the need for dialysis or transplantation in the SPRINT trial, and a composite occurrence of serum creatinine concentration >3.3 mg/dL, initiation of dialysis, or renal transplantation in the ACCORD-BP trial.

**†**Reductions in eGFR were defined as ≥50%, ≥40%, and ≥30% decline in eGFR levels from the baseline, and confirmed by a next available official laboratory test.

**‡**Incident albuminuria was defined by a doubling of UACR from a value <10 mg/g to a value of >10 mg/g; microalbuminuria was defined by UACR > 30 mg/g; macroalbuminuria was defined by UACR ratio > 300 mg/g. Albuminuria definitions were confirmed by a next available official laboratory test.

**§**Progression risk of CKD was classified based on eGFR and UACR according to KDIGO risk categories at baseline and follow-up period, and confirmed by a next available official laboratory test.

**#**Adjusted for baseline age, sex, race, history of cardiovascular disease, tobacco use, body-mass index, diabetes status, eGFR, UACR, SBP, and total cholesterol.

Abbreviations: CKD, chronic kidney disease; eGFR, estimated glomerular filtration rate; SBP, systolic blood pressure; UACR, urine albumin-to-creatinine ratio.

**Table S3 Effects of intensive vs. standard BP control on kidney outcomes in SPRINT participants with and without CKD during the intervention and post-intervention observational period**

|  | **Participants with CKD at baseline** | |  |  | **Participants without CKD at baseline** | |  |  |  |
| --- | --- | --- | --- | --- | --- | --- | --- | --- | --- |
|  | **Intensive** | **Standard** | **HR^#^**  **（95% CI）** | ***P* Value** | **Intensive** | **Standard** | **HR^#^**  **（95% CI）** | ***P* Value** | ***P* for interaction** |
|  | Events  (Events/1,000  person-years) | Events  (Events/1,000  person-years) |  |  | Events  (Events/1,000  person-years) | Events  (Events/1,000  person-years) |  |  |  |
| ***Renal failure** | 8 (1.65) | 13 (2.73) | 0.48 (0.17-1.31) | 0.152 | 0 | 0 | - | - | - |
| **†eGFR decline** |  |  |  |  |  |  |  |  |  |
| ≥30% Reduction | 124 (28.63) | 58 (13.63) | 2.10 (1.53-2.87) | <0.001 | 238 (21.96) | 79 (7.07) | 3.12 (2.42-4.02) | <0.001 | 0.089 |
| ≥40% Reduction | 39 (8.67) | 26 (6.02) | 1.36 (0.82-2.24) | 0.235 | 67 (5.98) | 22 (1.95) | 3.10 (1.91-5.02) | <0.001 | 0.053 |
| ≥50% Reduction | 18 (3.96) | 15 (3.45) | 1.14 (0.56-2.29) | 0.720 | 16 (1.42) | 9 (0.80) | 1.79 (0.79-4.08) | 0.166 | 0.520 |
| **‡Albuminuria** |  |  |  |  |  |  |  |  |  |
| Incident albuminuria | 70 (16.31) | 97 (23.93) | 0.67 (0.49-0.91) | 0.010 | 152 (14.23) | 198 (18.66) | 0.77 (0.63-0.96) | 0.017 | 0.439 |
| Microalbuminuria | 81 (18.87) | 129 (32.34) | 0.56 (0.43-0.75) | <0.001 | 106 (9.80) | 166 (15.52) | 0.63 (0.50-0.81) | <0.001 | 0.555 |
| Macroalbuminuria | 38 (8.68) | 56 (13.51) | 0.60 (0.40-0.91) | 0.017 | 8 (0.73) | 24 (2.19) | 0.32 (0.14-0.72) | 0.006 | 0.206 |
| **§CKD risk progression according to KDIGO risk categories** | | |  |  |  |  |  |  |  |
| Low-risk to moderate-risk | - | - | - | - | 386 (38.08) | 289 (27.87) | 1.46 (1.25-1.70) | <0.001 | - |
| Low-risk to high-risk | - | - | - | - | 36 (3.29) | 21 (1.92) | 1.83 (1.07-3.14) | 0.028 | - |
| Low-risk to very-high-risk | - | - | - | - | 2 (0.18) | 3 (0.27) | 0.70 (0.12-4.22) | 0.700 | - |
| Moderate-risk to high-risk | 124 (29.90) | 80 (19.70) | 1.52 (1.15-2.01) | 0.004 | 38 (3.48) | 42 (3.85) | 0.90 (0.58-1.40) | 0.632 | 0.048 |
| Moderate-risk to very-high-risk | 15 (3.40) | 11 (2.60) | 1.31 (0.60-2.87) | 0.492 | 4 (0.36) | 4 (0.36) | 1.00 (0.25-4.01) | 0.998 | 0.777 |
| High-risk to very-high-risk | 53 (12.26) | 36 (8.62) | 1.41 (0.92-2.16) | 0.111 | 6 (0.55) | 7 (0.64) | 0.41 (0.14-1.23) | 0.112 | 0.445 |

***** Renal failure was defined by the need for dialysis or transplantation in the SPRINT trial, and a composite occurrence of serum creatinine concentration >3.3 mg/dL, initiation of dialysis, or renal transplantation in the ACCORD-BP trial.

**†**Reductions in eGFR were defined as ≥50%, ≥40%, and ≥30% decline in eGFR levels from the baseline, and confirmed by a next available official laboratory test.

**‡**Incident albuminuria was defined by a doubling of UACR from a value <10 mg/g to a value of >10 mg/g; microalbuminuria was defined by UACR > 30 mg/g; macroalbuminuria was defined by UACR ratio > 300 mg/g. Albuminuria definitions were confirmed by a next available official laboratory test.

**§**Progression risk of CKD was classified based on eGFR and UACR according to KDIGO risk categories at baseline and follow-up period, and confirmed by a next available official laboratory test.

**#**Adjusted for baseline age, sex, race, history of cardiovascular disease, tobacco use, body-mass index, diabetes status, eGFR, UACR, SBP, and total cholesterol.

Abbreviations: CKD, chronic kidney disease; eGFR, estimated glomerular filtration rate; SBP, systolic blood pressure; UACR, urine albumin-to-creatinine ratio.

**Table S4 Effects of intensive vs. standard BP control on kidney outcomes in SPRINT eligible ACCORD-BP participants with and without CKD during the intervention period**

|  | **Participants with CKD at baseline** | |  |  | **Participants without CKD at baseline** | |  |  |  |
| --- | --- | --- | --- | --- | --- | --- | --- | --- | --- |
|  | **Intensive** | **Standard** | **HR^#^**  **（95% CI）** | ***P* Value** | **Intensive** | **Standard** | **HR^#^**  **（95% CI）** | ***P* Value** | ***P* for interaction** |
|  | Events  (Events/1,000  person-years) | Events  (Events/1,000  person-years) |  |  | Events  (Events/1,000  person-years) | Events  (Events/1,000  person-years) |  |  |  |
| ***Renal failure** | 3 (6.80) | 4 (11.27) | 0.44 (0.09-2.05) | 0.295 | 21 (4.75) | 21 (4.72) | 0.99 (0.54-1.83) | 0.986 | 0.416 |
| **†eGFR decline** |  |  |  |  |  |  |  |  |  |
| ≥30% Reduction | 15 (38.90) | 10 (29.79) | 1.28 (0.53-3.06) | 0.584 | 255 (69.66) | 130 (32.18) | 2.17 (1.76-2.69) | <0.001 | 0.292 |
| ≥40% Reduction | 5 (11.96) | 4 (11.56) | 0.87 (0.20-3.71) | 0.853 | 105 (25.63) | 56 (13.15) | 1.95 (1.40-2.71) | <0.001 | 0.438 |
| ≥50% Reduction | 3 (7.09) | 3 (8.58) | 0.27 (0.05-1.49) | 0.132 | 38 (8.89) | 21 (4.83) | 1.74 (1.02-2.97) | 0.043 | 0.466 |
| **‡Albuminuria** |  |  |  |  |  |  |  |  |  |
| Incident albuminuria | 3 (7.37) | 8 (24.54) | 0.72 (0.01-0.59) | 0.014 | 37 (8.81) | 45 (10.71) | 0.82 (0.53-1.27) | 0.372 | 0.103 |
| Microalbuminuria | 3 (7.39) | 9 (27.34) | 0.12 (0.02-0.59) | 0.009 | 48 (11.46) | 48 (11.43) | 0.97 (0.65-1.46) | 0.896 | 0.035 |
| Macroalbuminuria | 2 (4.88) | 8 (24.31) | 0.15 (0.03-0.84) | 0.031 | 15 (3.52) | 28 (6.61) | 0.49 (0.26-0.92) | 0.026 | 0.321 |
| **§CKD risk progression according to KDIGO risk categories** | | |  |  |  |  |  |  |  |
| Low-risk to moderate-risk | - | - | - | - | 91 (22.37) | 64 (15.40) | 1.42 (1.03-1.96) | 0.032 | - |
| Low-risk to high-risk | - | - | - | - | 20 (4.72) | 8 (1.87) | 2.41 (1.05-5.52) | 0.038 | - |
| Low-risk to very-high-risk | - | - | - | - | 3 (0.70) | 3 (0.70) | 0.85 (0.16-4.37) | 0.841 | - |
| Moderate-risk to high-risk | 12 (31.30) | 3 (8.75) | 4.17 (1.04-16.66) | 0.043 | 28 (6.64) | 34 (8.06) | 0.72 (0.44-1.20) | 0.209 | 0.012 |
| Moderate-risk to very-high-risk | 4 (9.87) | 1 (2.88) | 12.63 (0.46-348.50) | 0.134 | 6 (1.41) | 6 (1.40) | 1.08 (0.33-3.46) | 0.902 | 0.305 |
| High-risk to very-high-risk | 3 (7.45) | 6 (18.25) | 0.17 (0.03-0.81) | 0.027 | 11 (2.58) | 6 (1.40) | 1.78 (0.61-5.19) | 0.290 | 0.092 |

***** Renal failure was defined by the need for dialysis or transplantation in the SPRINT trial, and a composite occurrence of serum creatinine concentration >3.3 mg/dL, initiation of dialysis, or renal transplantation in the ACCORD-BP trial.

**†**Reductions in eGFR were defined as ≥50%, ≥40%, and ≥30% decline in eGFR levels from the baseline, and confirmed by a next available official laboratory test.

**‡**Incident albuminuria was defined by a doubling of UACR from a value <10 mg/g to a value of >10 mg/g; microalbuminuria was defined by UACR > 30 mg/g; macroalbuminuria was defined by UACR ratio > 300 mg/g. Albuminuria definitions were confirmed by a next available official laboratory test.

**§**Progression risk of CKD was classified based on eGFR and UACR according to KDIGO risk categories at baseline and follow-up period, and confirmed by a next available official laboratory test.

**#**Adjusted for baseline age, sex, race, history of cardiovascular disease, tobacco use, body-mass index, diabetes status, eGFR, UACR, SBP, and total cholesterol.

Abbreviations: CKD, chronic kidney disease; eGFR, estimated glomerular filtration rate; SBP, systolic blood pressure; UACR, urine albumin-to-creatinine ratio.

**Table S5 Effects of intensive vs. standard BP control on kidney outcomes in SPRINT eligible ACCORD-BP participants with and without CKD during the intervention and post-intervention observational period**

|  | **Participants with CKD at baseline** | |  |  | **Participants without CKD at baseline** | |  |  |  |
| --- | --- | --- | --- | --- | --- | --- | --- | --- | --- |
|  | **Intensive** | **Standard** | **HR^#^**  **（95% CI）** | ***P* Value** | **Intensive** | **Standard** | **HR^#^**  **（95% CI）** | ***P* Value** | ***P* for interaction** |
|  | Events  (Events/1,000  person-years) | Events  (Events/1,000  person-years) |  |  | Events  (Events/1,000  person-years) | Events  (Events/1,000  person-years) |  |  |  |
| ***Renal failure** | 4 (5.61) | 9 (15.61) | 0.18 (0.05-0.61) | 0.006 | 27 (3.47) | 29 (3.82) | 0.88 (0.52-1.50) | 0.651 | 0.089 |
| **†eGFR decline** |  |  |  |  |  |  |  |  |  |
| ≥30% Reduction | 16 (32.01) | 14 (32.83) | 0.95 (0.43-2.10) | 0.908 | 296 (59.61) | 174 (31.27) | 1.93 (1.59-2.33) | <0.001 | 0.101 |
| ≥40% Reduction | 6 (10.99) | 8 (17.97) | 0.51 (0.16-1.63) | 0.256 | 134 (23.09) | 79 (13.16) | 1.78 (1.34-2.36) | <0.001 | 0.074 |
| ≥50% Reduction | 3 (5.35) | 5 (11.00) | 0.19 (0.04-0.83) | 0.027 | 46 (7.46) | 33 (5.34) | 1.36 (0.86-2.16) | 0.187 | 0.210 |
| **‡Albuminuria** |  |  |  |  |  |  |  |  |  |
| Incident albuminuria | 5 (9.31) | 11 (26.45) | 0.16 (0.03-0.71) | 0.016 | 72 (12.08) | 69 (11.70) | 0.99 (0.71-1.38) | 0.941 | 0.048 |
| Microalbuminuria | 7 (13.31) | 10 (23.67) | 0.37 (0.12-1.08) | 0.069 | 77 (12.96) | 69 (11.71) | 1.06 (0.76-1.47) | 0.743 | 0.104 |
| Macroalbuminuria | 6 (11.16) | 9 (21.74) | 0.43 (0.14-1.35) | 0.147 | 35 (5.73) | 36 (5.95) | 0.91 (0.57-1.46) | 0.704 | 0.337 |
| **§CKD risk progression according to KDIGO risk categories** | | |  |  |  |  |  |  |  |
| Low-risk to moderate-risk | - | - | - | - | 119 (20.94) | 86 (14.87) | 1.38 (1.04-1.82) | 0.025 | - |
| Low-risk to high-risk | - | - | - | - | 36 (5.91) | 14 (2.29) | 2.48 (1.32-4.64) | 0.005 | - |
| Low-risk to very-high-risk | - | - | - | - | 7 (1.13) | 8 (1.30) | 0.88 (0.31-2.51) | 0.810 | - |
| Moderate-risk to high-risk | 14 (27.26) | 5 (11.46) | 2.21 (0.73-6.72) | 0.162 | 38 (6.28) | 40 (6.67) | 0.85 (0.54-1.33) | 0.482 | 0.044 |
| Moderate-risk to very-high-risk | 6 (11.08) | 2 (4.47) | 4.52 (0.68-29.88) | 0.118 | 14 (2.34) | 13 (2.17) | 1.06 (0.49-2.30) | 0.876 | 0.271 |
| High-risk to very-high-risk | 6 (11.32) | 6 (14.18) | 0.68 (0.20-2.29) | 0.529 | 14 (2.27) | 14 (2.30) | 1.07 (0.48-2.35) | 0.876 | 0.869 |

***** Renal failure was defined by the need for dialysis or transplantation in the SPRINT trial, and a composite occurrence of serum creatinine concentration >3.3 mg/dL, initiation of dialysis, or renal transplantation in the ACCORD-BP trial.

**†**Reductions in eGFR were defined as ≥50%, ≥40%, and ≥30% decline in eGFR levels from the baseline, and confirmed by a next available official laboratory test.

**‡**Incident albuminuria was defined by a doubling of UACR from a value <10 mg/g to a value of >10 mg/g; microalbuminuria was defined by UACR > 30 mg/g; macroalbuminuria was defined by UACR ratio > 300 mg/g. Albuminuria definitions were confirmed by a next available official laboratory test.

**§**Progression risk of CKD was classified based on eGFR and UACR according to KDIGO risk categories at baseline and follow-up period, and confirmed by a next available official laboratory test.

**#**Adjusted for baseline age, sex, race, history of cardiovascular disease, tobacco use, body-mass index, diabetes status, eGFR, UACR, SBP, and total cholesterol.

Abbreviations: CKD, chronic kidney disease; eGFR, estimated glomerular filtration rate; SBP, systolic blood pressure; UACR, urine albumin-to-creatinine ratio.
